# Transcriptomic-Based Identification of the Immuno-Oncogenic Signature of Cholangiocarcinoma for HLC-018 Multi-Target Therapy Exploration

**DOI:** 10.3390/cells10112873

**Published:** 2021-10-25

**Authors:** Bashir Lawal, Yu-Cheng Kuo, Sung-Ling Tang, Feng-Cheng Liu, Alexander T. H. Wu, Hung-Yun Lin, Hsu-Shan Huang

**Affiliations:** 1 PhD Program for Cancer Molecular Biology and Drug Discovery, College of Medical Science and Technology, Taipei Medical University and Academia Sinica, Taipei 11031, Taiwan; bashirlawal12@gmail.com; 2 Graduate Institute of Cancer Biology & Drug Discovery, College of Medical Science and Technology, Taipei Medical University, Taipei 11031, Taiwan; 3 Department of Pharmacology, School of Medicine, College of Medicine, Taipei Medical University, Taipei 11031, Taiwan; yuchengkuo@tmu.edu.tw; 4 School of Post-baccalaureate Chinese Medicine, College of Chinese Medicine, China Medical University, Taichung 40402, Taiwan; 5 Department of Pharmacy Practice, Tri-Service General Hospital, School of Pharmacy, National Defense Medical Center, Taipei 11490, Taiwan; tasuling@gmail.com; 6 Department of Rheumatology/Immunology and Allergy, Department of Medicine, Tri-Service General Hospital, National Defense Medical Center, Taipei 114, Taiwan; lfc10399@yahoo.com.tw; 7 The PhD Program of Translational Medicine, College of Medical Science and Technology, Taipei Medical University, Taipei 11031, Taiwan; 8 Clinical Research Center, Taipei Medical University Hospital, Taipei Medical University, Taipei 11031, Taiwan; 9 TMU Research Center of Cancer Translational Medicine, Taipei Medical University, Taipei 11031, Taiwan; 10 Traditional Herbal Medicine Research Center of Taipei Medical University Hospital, Taipei Medical University, Taipei 11031, Taiwan; 11 Graduate Institute of Medical Sciences, National Defense Medical Center, Taipei 11490, Taiwan; 12 PhD Program in Drug Discovery and Development Industry, College of Pharmacy, Taipei Medical University, Taipei 11031, Taiwan

**Keywords:** cholangiocarcinoma, tumor microenvironment, therapeutic resistance, receptor-ligand interaction, molecular docking, CHOL-hub gene

## Abstract

Cholangiocarcinomas (CHOLs), hepatobiliary malignancies, are characterized by high genetic heterogeneity, a rich tumor microenvironment, therapeutic resistance, difficulty diagnosing, and poor prognoses. Current knowledge of genetic alterations and known molecular markers for CHOL is insufficient, necessitating the need for further evaluation of the genome and RNA expression data in order to identify potential therapeutic targets, clarify the roles of these targets in the tumor microenvironment, and explore novel therapeutic drugs against the identified targets. Consequently, in our attempt to explore novel genetic markers associated with the carcinogenesis of CHOL, five genes (*SNX15*, *ATP2A1*, *PDCD10*, *BET1*, and *HMGA2*), collectively termed CHOL-hub genes, were identified via integration of differentially expressed genes (DEGs) from relatively large numbers of samples from CHOL GEO datasets. We further explored the biological functions of the CHOL-hub genes and found significant enrichment in several biological process and pathways associated with stem cell angiogenesis, cell proliferation, and cancer development, while the interaction network revealed high genetic interactions with a number of onco-functional genes. In addition, we established associations between the CHOL-hub genes and tumor progression, metastasis, tumor immune and immunosuppressive cell infiltration, dysfunctional T-cell phenotypes, poor prognoses, and therapeutic resistance in CHOL. Thus, we proposed that targeting CHOL-hub genes could be an ideal therapeutic approach for treating CHOLs, and we explored the potential of HLC-018, a novel benzamide-linked small molecule, using molecular docking of ligand-receptor interactions. To our delight, HLC-018 was well accommodated with high binding affinities to binding pockets of CHOL-hub genes; more importantly, we found specific interactions of HLC-018 with the conserved sequence of the AT-hook DNA-binding motif of *HMGA2*. Altogether, our study provides insights into the immune-oncogenic phenotypes of CHOL and provides valuable information for our ongoing experimental validation.

## 1. Introduction

Cholangiocarcinomas (CHOLs) are hepatobiliary malignancies ranked as the 2nd most prevalent hepatic cancer next to hepatocellular carcinoma [1]. They are characterized by cholangiocyte phenotypic features and are anatomically classified into intrahepatic, perihilar, and distal CHOLs based on their respective locations in the periphery of second-order bile ducts, the right and/or left hepatic duct, and the common bile duct [2]. Percentage occurrences of the perihilar, distal, and intrahepatic types of CHOLs are 50%, 42%, and 8%, respectively [3]. These subtypes also differ in their etiology, epidemiology, pathogenesis, diagnosis, and treatment [4]. However, these subtypes are united by a late diagnosis, limited curative options, and poor prognoses [5]; they all have a median survival of 24 months after diagnosis [4].

Major risk factors for CHOLs include cirrhosis, hepatitis B virus (HBV), hepatitis C virus (HCV), liver flukes, toxins, excessive alcohol intake, and metabolic disorders such as diabetes, obesity, and nonalcoholic fatty liver disease [6,7]. These risk factors, however, greatly vary by geographic area. Although recent years have witnessed the emergence of novel treatment targets, medical therapy remains a compelling challenge in hepatobiliary malignancies [8,9,10]. The only curative treatment option for early-stage disease is surgery [11], and no conclusive evidence for the efficacy of chemotherapy has been documented [5]. The heterogenic complexity of CHOLs supported by the rich tumor microenvironment (TME) is a major contributor to high therapeutic failure [3]. Furthermore, dynamic regulatory mechanisms of interactions between the stromal and immune components of the TME in the progression of CHOL remain poorly understood.

Accumulating evidence has established that abnormal gene expression profiles are frontline etiologic factors in the carcinogenesis and progression of human cancers. However, considering the heterogeneity of CHOLs, current knowledge on genetic alterations and known molecular markers is insufficient, necessitating the need for further evaluation of genomic and RNA expression data of CHOLs to identify potential therapeutic targets, clarify the roles of those targets in modulating the immunophenotypes of tumors, and explore novel therapeutic drugs against the identified targets [12]. A myriad of large-scale human cancer genomics projects offer large quantities of genomic and clinical data of cancer patients to analyze prognostic-relevant differentially expressed genes (DEGs) and identify biomarkers of disease progression and therapeutic targets [13].

Herein, we integrated DEGs from relatively large CHOL datasets obtained from the Gene Expression Omnibus (GEO) to identify the most implicated genes (which we refer to as CHOL-hub genes) in the development of CHOLs. We further explored the biological functions of these DEGs and constructed a protein-protein interaction (PPI) network. In addition, we established associations between the CHOL-hub genes and tumor progression, metastasis, tumor immune and immunosuppressive cell infiltration, poor prognoses, and therapeutic resistance in CHOLs.

In our previous studies, a myriad of in-house-synthesized [14,15,16] multi-target small molecules were explored for preclinical efficacy in attenuating cell proliferation, therapeutic resistance, and aggressive phenotypes of various cancers [17,18,19,20,21,22,23,24]. Furthermore, these small molecules have demonstrated promising activities in the treatment of osteoarthritis [25,26]. In our ongoing efforts to obtain derivatives of great potency of these compounds, in the present study, we report a novel benzamide-linked small molecule, 6-(2,4-difluorophenyl)-3-(3-(trifluoromethyl)phenyl)-2H-benzo[e][1,3]oxazine-2,4(3H)-dione (HLC-018), and explored its potential as a therapeutic target against CHOL-hub genes through an in silico ligand-receptor interaction study. To our delight, HLC-018 was well accommodated with high binding affinities to the binding pockets of CHOL-hub genes, and more importantly, we found specific interactions of HLC-018 with the conserved sequence of the AT-hook DNA-binding motif of *HMGA2*. Altogether, our study provides insights into the immune-oncogenic phenotypes of CHOLs and provides valuable information for ongoing experimental validation.

## 2. Methods

### 2.1. Transcriptome Data Acquisition and DEG Identification

We mined the NCBI GEO, a public functional genomics data repository to obtain high-throughput sequencing and microarray-based gene expression profile data from five CHOL datasets (GSE132305, GSE31370, GSE38860, GSE45001, and GSE32225). The datasets consisted of gene expression profiles from CHOL patients vs. control cohorts. Detailed characteristics of the datasets are presented in Table 1. GEO2R tools were adopted to screen for DEGs from each dataset. The obtained DEGs were further screened to identify the most significant ones using the limma R package with a threshold of|log_2_multiple of change|of >1.5 and *p* < 0.05. DEGs from each dataset were superimposed to identify common intersecting DEGs, which were visualized using the Multiple List Comparator web tool.

### 2.2. Differential Expressions of CHOL-Hub Genes among Tumor Grades and with Nodal Metastasis

We analyzed differential expression levels of CHOL-hub genes between tumor and adjacent normal tissues of The Cancer Genome Atlas (TCGA) CHOL dataset using the Tumor Immune Estimation Resource (TIMER, vers. 2.0) [27]. We downloaded transcript expression levels of CHOL-hub genes in CHOL patients from TCGA cancer database via the UALCAN server [28] and compared expression levels of CHOL-hub genes among four types of tumor grades, including well-differentiated (low grade, grade 1), moderately differentiated (intermediate grade, grade 2), poorly differentiated (high grade, grade 3), and undifferentiated (high grade, grade 4). We also compared expression profiles of CHOL-hub genes between nodal metastasis statuses by comparing transcript expression levels of CHOL-hub genes between CHOL patients with no regional lymph node metastasis (N0) and patients with metastases in one to three axillary lymph nodes (N1). Differential expression was considered statistically significance at *p* < 0.05, <0.01, and <0.001.

### 2.3. Prognostic Analysis of CHOL-Hub Genes

Survival times (in days) and RNA expression data of CHOL-hub genes from tumor samples collected from CHOL patients in TCGA datasets were downloaded from the GDC portal using the Q-omics algorithm (wndows, vers. 0.9). Patients were split into high- and low-expression groups based on median expression levels of CHOL-hub genes. Overall survival (OS) and disease-free survival (DFS) of cohorts in each group were computed with a hazard ratio (HR), a 95% confidence interval (CI), and a log-rank test *p* value. In addition, we used the SurvExpress algorithm [29] to evaluate survival gene expression data of CHOL-hub genes using the CHOL-TCGA CHOL dataset, consisting of 35 CHOL patients. Survival times (in days) of the cohorts were censored and compared between cohorts with higher expression levels and those with lower expression levels of CHOL-hub genes. A risk index was computed, and hazard ratios (HRs) were estimated by fitting a CoxPH using the risk group as a covariate.

### 2.4. Functional Enrichment and Interaction Network Analysis of CHOL-Hub Genes

The online gene set enrichment analysis (GSEA) server, Enrichr [30,31], was used to analyze functional enrichment profiles of CHOL-hub genes based on Kyoto Encyclopedia of Genes and Genomes (KEGG) pathways and gene ontology (GO) terms of biological processes. Enrichment terms were considered significant at *p* < 0.05, and visualization was achieved using the R package cluster Profiler [32]. The interaction analysis and network construction for gene-gene interactions (GGIs) of CHOL-hub genes were performed using GeneMANIA, a real-time multiple association network integration algorithm for predicting gene functions [33].

### 2.5. Analysis of CHOL-Hub Gene Associations with Drug Sensitivity

We downloaded the area under the dose-response curve (AUC) values for anticancer small molecules and CHOL-hub gene expression profiles in different cancer cell lines from the drug-cell response sensitivity repository data of the Genomics of Drug Sensitivity in Cancer (GDSC) and CTRP through the GSCALite server [34]. We then used Spearman correlation coefficients to analyze correlations between CHOL-hub gene expression levels and drug sensitivity (50% inhibitory concentration (IC_50_)) to 265 small molecules from the GDSC and CTRP databases. The top 30 most correlated compounds are presented in a bubble plot.

### 2.6. Analysis of CHOL-Hub Genes’ Associations with the TME and Immunophenotypes in CHOL

We used the ImmuCellAI (Immune Cell Abundance Identifier) algorithm to estimate the tumor infiltration of 24 immune cells including 18 T-cell subtypes and six other immune cells (natural killer (NK) cells, monocytes, macrophages, neutrophils, dendritic cells (DCs), and B cells) from RNA-sequencing (Seq) data of CHOL-hub genes. A correlation analysis was conducted using purity-corrected partial Spearman’s rho values and statistical significance based on *p* values of the Wilcoxon test and the false discovery rate (FDR). Data visualization was done using the GSCALite online server [34]. In addition, we used the QUERY module of the TIDE algorithm to evaluate the effect of CHOL-hub gene methylation on dysfunctional T-cell phenotypes [35].

### 2.7. Molecular Docking Studies of CHOL-Hub Genes with HLC-018

Crystal structures of CHOL-hub genes of *SNX15* (PDB:6ECM), *ATP2A1* (PDB:4H1W), *PDCD10* (PDB:3AJM), *BET1* (PDB:3EGX), and *HMGA2* (PDB:3UXW) in PDB file format were obtained from the Protein Data Bank, while the three-dimensional (3D) structure of LCC18 in mol2 format was obtained using the Avogadro molecular builder and visualization tool vers. 1.XX [36]. These were converted to PDB files using the PyMOL Molecular Graphics System, vers. 1.2r3pre. All PDB files were subsequently converted to PDBQT files using AutoDock Vina (vers. 0.8, Scripps Research Institute, La Jolla, CA, USA) [37]. Each of the CHOL-hub gene targets was prepared for docking by removing water molecules and adding polar hydrogen atoms and charges [38,39,40]. Docking was conducted using AutoDock Vina as described in previous studies [38,41], and results were visualized using the PyMOL, Discovery studio visualizer vers. 19.1.0.18287 (BIOVIA, San Diego, CA, USA) [42] and protein-ligand interaction profiler web tool [43].

## 3. Results

### 3.1. SNX15, ATP2A1, PDCD10, BET1, and HMGA2 Are Biomarkers of Tumor Progression and Metastasis in CHOL Patients

**Figure 1:**
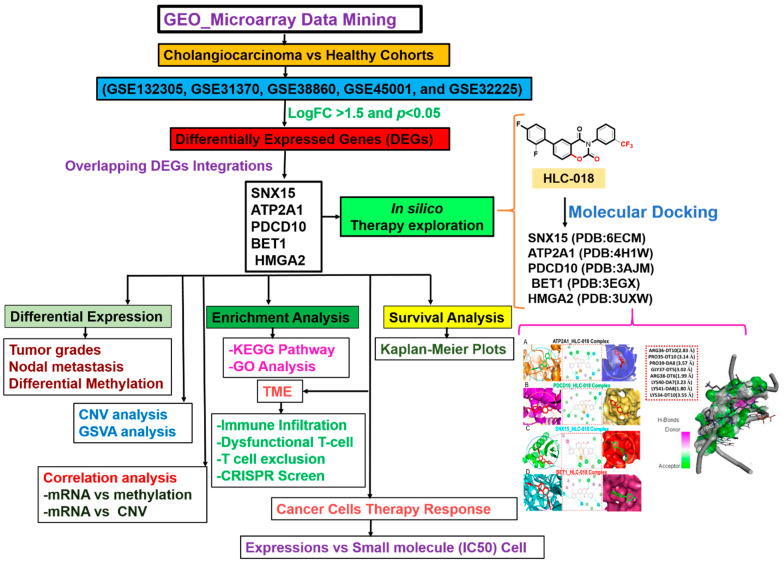
Schematic flow chart of the study design for identifying and characterizing the pathological role of DEGs in cholangiocarcinoma and in silico exploration of HLC-018 as a potential therapeutic candidate.

**Figure 2:**
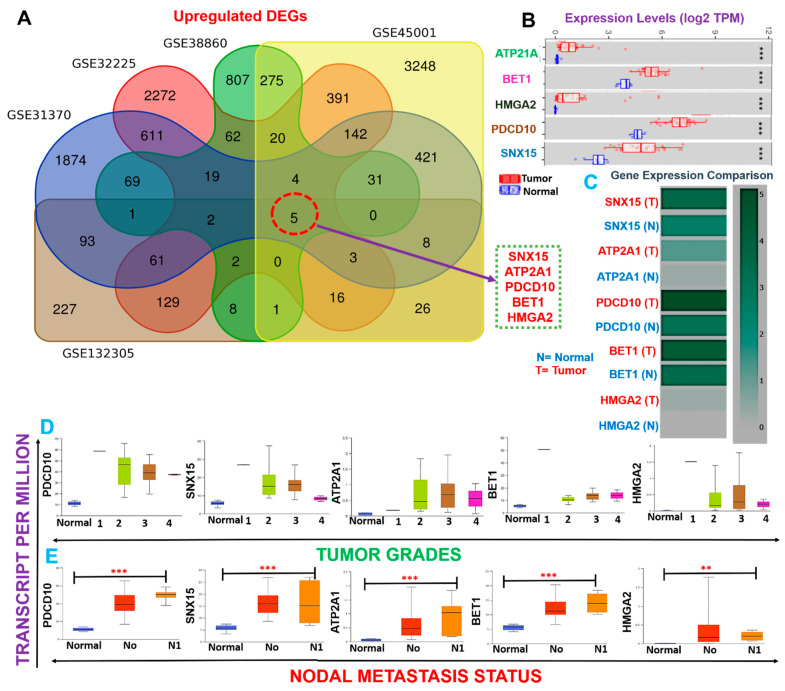
Cholangiocarcinoma (CHOL)-hub genes are biomarkers of tumor progression and metastasis in CHOL patients. (**A**) Venn diagram showing the distribution of differentially expressed genes (DEGs) in each dataset and overlapping upregulated DEGs. (**B**) Boxplot showing differential mRNA expression levels of CHOL-hub genes between TCGA CHOL tumor samples and adjacent normal controls. (**C**) Heatmap of expression difference among CHOL-hub genes. (**D**) Box plot showing differential expression levels of hub genes among tumor grades (**E**) and nodal metastasis statuses in TCGA cohorts of CHOL. ** *p* < 0.01, *** *p* < 0.001.

A schematic flow chart summarizing the overall study design for the identification and evaluation of the pathological role of DEGs in cholangiocarcinoma is shown in Figure 1. With the aim of identifying biomarker hub genes associated with the etiology and progression of CHOLs, we integrated DEGs from five microarray datasets of CHOL cohorts vs. normal patients (Table 1, Figure 1) and identified *SNX15*, *ATP2A1*, *PDCD10*, *BET1*, and *HMGA2* as the only overlapping DEGs across the five datasets (Figure 2A). Furthermore, we analyzed differential expression profiles of CHOL-hub genes between cancer and matched normal tissues across all TCGA tumors and found that CHOL cancer tissues expressed *SNX15*, *ATP2A1*, *PDCD10*, *BET1*, and *HMGA2* at significantly (*p* < 0.001) higher levels than did adjacent normal tissues (Figure 2B). Gene expression comparisons revealed that expression levels of hub genes in CHOL patients occurred in a significant order of *PDCD10* > *BET1* > *SNX15* > *ATP2A1* > *HMGA2* (Figure 2C). However, we compared expression profiles between tumor grades and found that *SNX15*, *ATP2A1*, *PDCD10*, *BET1*, and *HMGA2* expressions were significantly (all *p* < 0.05) associated with tumor stages (Figure 2D). In addition, we found that CHOL patients with metastases in one to three axillary lymph nodes exhibited significantly (all *p* < 0.05) higher expression levels of *SNX15*, *ATP2A1*, *PDCD10*, *BET1*, and *HMGA2* than patients with no regional lymph node metastasis (Figure 2E). Suggestively, our results indicated that *SNX15, ATP2A1, PDCD10, BET1,* and *HMGA2* could serve as a biomarker signature of tumor progression and metastasis in CHOL patients.

### 3.2. SNX15, ATP2A1, PDCD10, BET1, and HMGA2 Are Associated with Higher Risks and Poor Prognoses of CHOL

**Figure 3:**
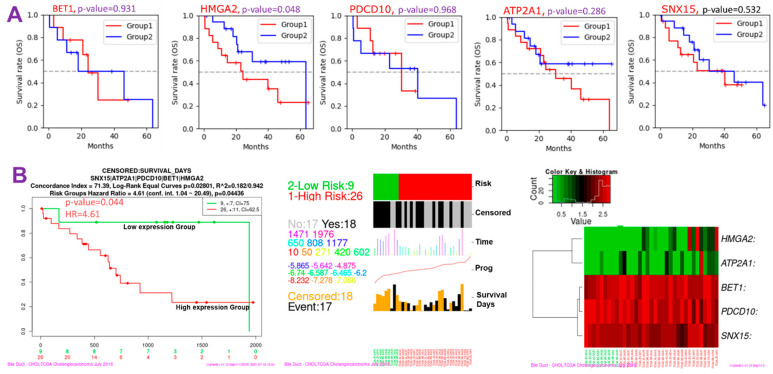
Cholangiocarcinoma (CHOL)-hub genes are associated with higher risks and poorer prognoses of CHOL. (**A**) Kaplan–Meier survival plot (**B**) Kaplan–Meier plots and hazard plots of CHOL-hub genes in CHOL patients.

Since CHOL-hub genes were found to be overexpressed and associated with tumor stages and metastasis (*p* < 0.05) in CHOL patients, we queried whether these genes could individually predict the survival of CHOL patients. Interestingly we found that out of the five hub genes, only *HMGA2* (HR = 1.914, *p* = 0.048) demonstrated an independent significant association with a worse prognosis of CHOL patients (Figure 3A, Supplementary Table S1). Furthermore, we queried the collective survival effect of CHOL-hub genes and found that higher expression levels of CHOL-hub genes were significantly associated with higher risk and achieved significant worse prognoses (HR = 4.61, *p* = 0.044) of CHOL patients (Figure 3B). However, our multivariable and HR analysis revealed that the differential expression levels of the CHOL hub genes achieved no significant (*p* > 0.05) hazard risk among the tumor stages (Supplementary Table S1).

### 3.3. CHOL-Hub Genes Potentially Promote Tumor Progression by Crosstalking with Multiple Oncogenic Targets/Pathways

**Figure 4:**
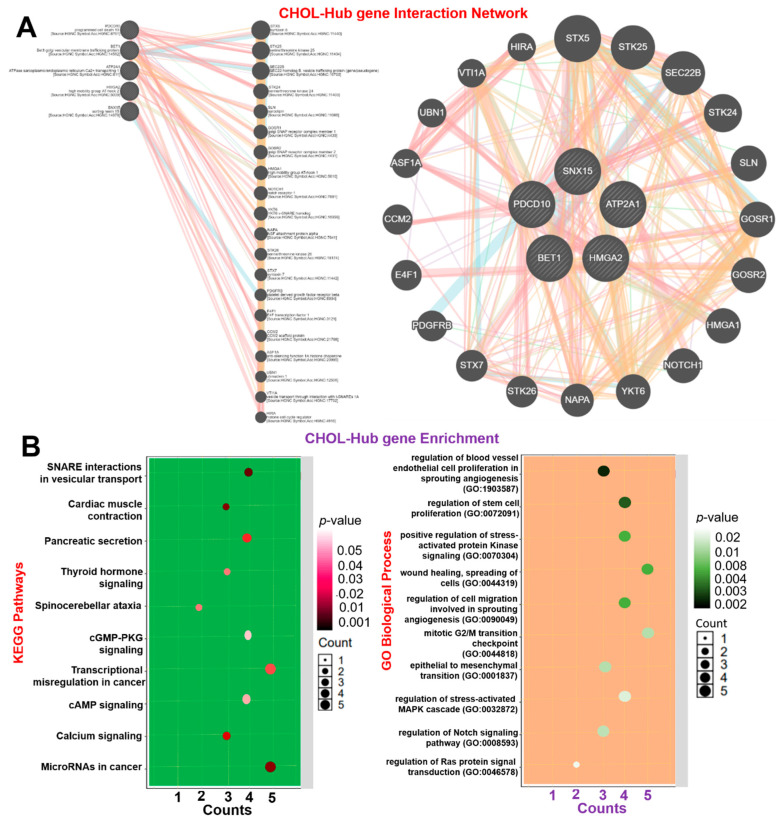
Gene interaction and enrichment analysis of cholangiocarcinoma (CHOL)-hub genes and (**A**) gene-gene interaction (GGI) network. (**B**) Enrichment of KEGG pathways (left panel) and GO biological processes (right panel) of hub genes.

Deciphering genetic interactions is vital to understanding cellular and organismal responses to gene-level alterations in cancer. Under the hypothesis that functionally related genes tend to share common genetic interaction partners [44], we evaluated GGIs of CHOL-hub genes and observed high genetic interactions with a number of onco-functional genes, including *STX5*, *STK25*, *SEC22B*, *STK24*, *SLN*, *GOSR1*, *GOSR2*, *HMGA1*, *NOTCH1*, *YKT6*, *NAPA*, *STK26*, *STX7*, *PDGFRB*, *E4F1*, *CCM2*, *ASF1A*, *UBN1*, *VTI1A*, and *HIRA* (Figure 4A). Interestingly, the CHOL-hub genes exhibited coexpression interactions with the expression levels of the onco-functional genes (Supplementary Table S2). Additionally, we analyzed the KEGG and GO enrichment of hub genes and found significant enrichment in several biological processes and pathways associated with stem cell angiogenesis, cell proliferation, and cancer development (Figure 4B). Enriched KEGG pathways of hub genes were related to SNARE interactions in vesicular transport, cardiomyopathy, pancreatic secretion, microRNAs in cancer, transcriptional misregulation in cancer, thyroid hormone signaling pathway, calcium signaling pathway, cGMP-PKG signaling pathway, and cAMP signaling pathway. GO biological processes in which hub genes were enriched included regulation of blood vessel endothelial cell proliferation involved in sprouting angiogenesis, regulation of stem cell proliferation, positive regulation of stress-activated protein kinase signaling cascade, wound healing, spreading of cells, regulation of cell migration involved in sprouting angiogenesis, mitotic G_2_/M transition checkpoint, and the epithelial-to-mesenchymal transition (EMT). Collectively, our results suggested that the CHOL-hub genes promote tumor progression by crosstalking with multiple oncogenic pathways and thus provides a rationale for targeting these genes as a therapeutic strategy to curb the activities of the multiple oncogenic pathways. 

### 3.4. CHOL-Hub Genes Are Potential Drivers of Invasive Immunophenotypes of CHOL

Expression levels of *HMGA2* and *PDCD10* in CHOL were significantly correlated with tumor infiltration by seven and nine immune cell types, respectively. *HMGA2* showed negative correlations (r = −0.30~−0.48, *p* < 0.05) with tumor infiltration by Th17 cells, monocytes, and macrophages and positive correlations (r = 0.33~−0.57, *p* < 0.05) with tumor infiltration of B cells, NKT cells, nTregs, and CD8-naive cells. *PDCD10* expression was negatively correlated (r = −0.33~−0.43, *p* < 0.05) with tumor infiltration by MAIT, NK cells, and monocytes, and positively correlated (r = 0.29~−0.51, *p* < 0.05) with tumor infiltration by B cells, CD4_T cells, DCs, iTregs, nTregs, and Tr1s. Expression levels of *ATP2A1* were negatively associated with monocytes and macrophages, and positively correlated with B-cell infiltration, while *BET1* demonstrated positive correlations only with tumor infiltration by macrophages and Th1 cells in CHOL (Table 2).

**Figure 5:**
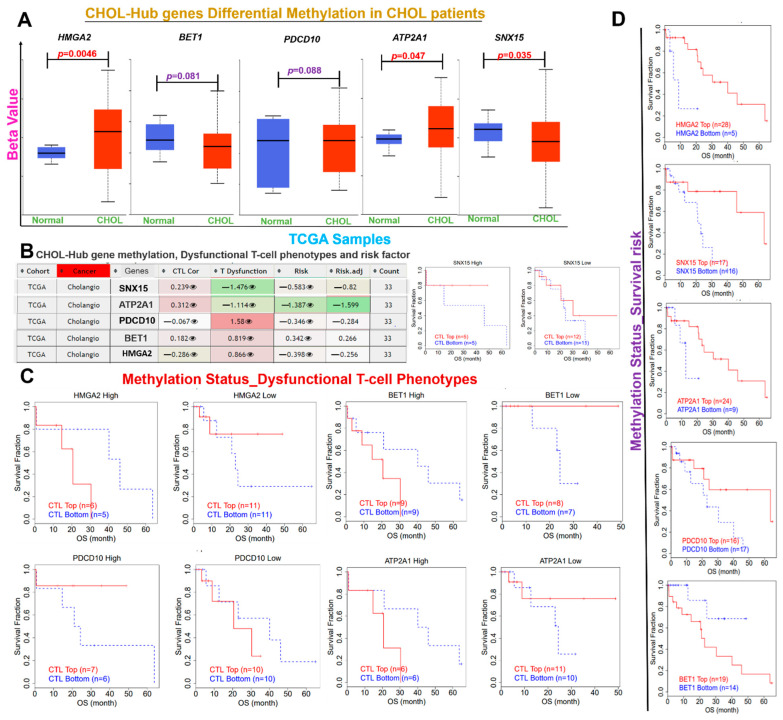
Differential Methylation of CHOL-Hub gene is associated with Dysfunctional T-cell Phenotypes in CHOL patients. (**A**) Boxplot showing differential methylation levels of CHOL-hub genes between TCGA CHOL tumor samples and healthy. (**B**) Heatmap of CHOL-Hub gene methylation and its association with Dysfunctional T-cell phenotypes and risk factor of CHOL patients. (**C**) Kaplan–Meier plot showing the association between the methylation status of the CHOL-hub gene and dysfunctional T-cell phenotypes of CHOL patient (**D**) Kaplan–Meier plot showing the association between the methylation status of the CHOL-hub gene and survival risk of CHOL patient.

We queried the role of methylation and CNV of the CHOL-hub genes in mediating the invasive immunophenotypes of CHOL patients using the TCGA cohorts (Figure 5A–D). We found that HMGA2 and ATP2A1 are significantly (*p* < 0.05) hypermethylated, while SNX15 is hypomethylated in CHOL patients when compared with the normal cohort (Figure 5A). The differential methylation of CHOL-hub genes predicted dysfunctional T-cell phenotypes (Figure 5B,C) and a high survival risk of the cohorts (Figure 5D).

**Figure 6:**
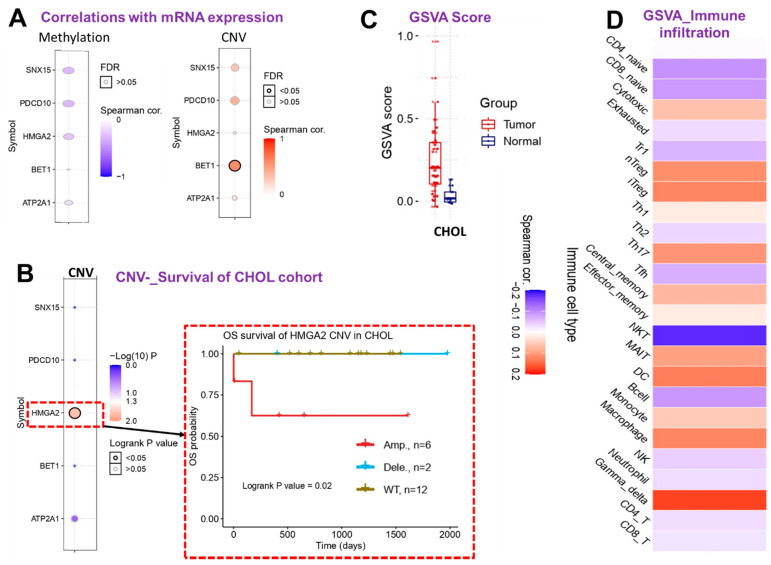
Cholangiocarcinoma (CHOL)-hub gene associations with tumor immune and immunosuppressive cell infiltration in CHOL patients. (**A**) Bubble plot showing correlations of the mRNA expression levels of CHOL-hub genes with methylation and copy number variation (CNV). (**B**) Heat map plot and Kaplan–Meier plot showing survival differences between CHOL patient with CNV of CHOL-hub genes and cohorts with wild-type genes. (**C**) Box plot showing GSVA scores of CHOL-hub genes between CHOL tumor and adjacent normal samples. (**D**) Heatmap showing correlations between GSVA scores of CHOL-hub genes and infiltration levels of various cells in CHOL patients.

However, our exploration of correlations between mRNA expressions and the methylation or CNV of CHOL-hub genes in CHOL patients revealed no significant associations except for the positive correlation between mRNA expression and CNV of *BET1* (Figure 6A). Survival analysis revealed that out of the five CHOL-hub genes, only *HMGA2* exhibited CNV (amplification) and was significantly (*p* = 0.02) associated with a worse prognosis compared to patients with wide-type *HMGA2* (Figure 6B). We queried the collective effect of the entire CHOL-hub gene set on tumor infiltrations of immune cells, and found that a higher GSVA score of CHOL-hub genes in CHOL tumor samples (Figure 6C) was positively correlated with higher tumor infiltrations of nTregs, iTregs, Th17 cells, central memory, MAIT, DCs, monocytes, macrophages, and Gamma-delta cells in CHOL patients, while negative corrections were observed between high GSVA scores of CHOL-hub genes and tumor infiltration by NKT, B-cells, CD4 and CD8, Tr1, and Tfh (Figure 6D). Collectively, our results suggested that the CHOL-hub genes are associated with tumor immune infiltrations and are potential drivers of dysfunctional T cell and invasive immunophenotypes of CHOL. We explored a single-cell RNA-sequencing dataset to evaluate which type of cells actually expressed the CHOL-hub genes. Our results revealed that *SNX15, ATP2A1, PDCD10, BET1, HMGA2* are expressed by the T cells; however, only the BET1 and *PDCD10* are expressed by the B cells (Supplementary Figure S1).

### 3.5. The CHOL Hub Genes Are Associated with Worse Immune-Oncological Phenotypes and Therapy Outcomes

**Figure 7:**
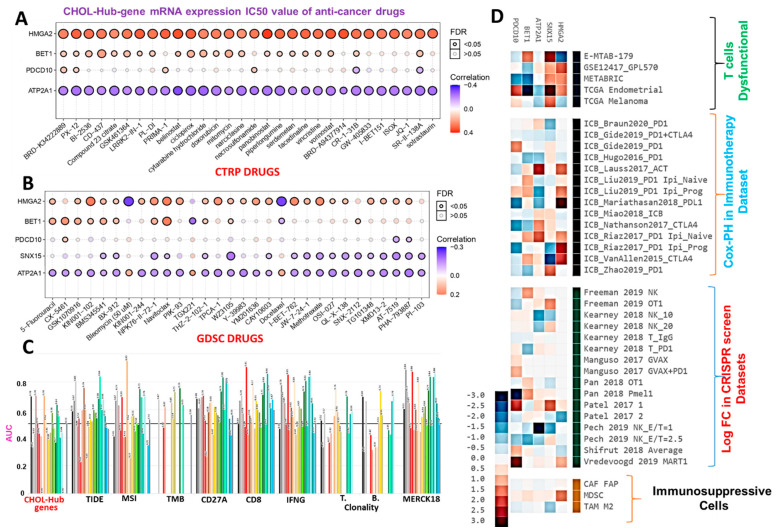
Cholangiocarcinoma (CHOL)-hub genes exhibited different associations with the sensitivity of cancer cells to anticancer therapy. Bubble plot of correlations of mRNA expression levels of hub genes with (**A**) CTRP and (**B**) GDSC drug sensitivities. Colors from blue to red represent correlations between mRNA expressions and IC_50_ values of small-molecule drugs. A positive correlation means that the gene’s high expression was resistant to the drug and vice versa. The bubble size was positively correlated with the false discovery rate (FDR) significance. The black outline border indicates an FDR of <0.05. (**C**) Bar plot of the comparative biomarker relevance between the CHOL-Hub genes and standardized biomarkers. (**D**) Heat map depicting the association between the CHOL hub genes and outcomes of ICB therapy, T-cell dysfunctional and exclusion markers, and T cell-mediated tumor killing in CRISPR screens, CAFs; cancer-associated fibroblasts; MDSCs; myeloid-derived suppressor cells; M2-TAMs; M2 subtype of tumor-associated macrophages.

We evaluated the effects of CHOL-hub genes on anticancer drug sensitivity by analyzing associations between mRNA expression levels of CHOL-hub genes and IC_50_ values of anticancer small molecules against various cancer cell lines using the drug-cell response sensitivity repository data of the GDSC and CTRP. Interestingly, we found that out of the top 30 compounds for which data were explored from CTRP, *HMGA2* demonstrated strong positive associations, while *ATP2A1* demonstrated strong negative associations with IC_50_ values of all compounds, suggesting sharp contrasts in the roles of the former compared to the latter (Figure 7A). Similarly, IC_50_ values of most of the small molecules from the GDSC database showed positive correlations with mRNA expression levels of *BET1* and *HMGA2*, while *ATP2A1,* in contrast to *BET1* and *HMGA2*, demonstrated negative associations with IC_50_ values of the drugs (Figure 7B). In order to gain a generalized insight of the biomarker relevance and role of the hub genes, we compared the predictive value of the CHOL-Hub genes with 8 standardized biomarkers and accessed the gene prioritization of the CHOL-hub genes in various immune cohorts’ datasets, including the ICB therapy outcome, dysfunctional or exclusion T-cell phenotypes, and datasets of T-cell mediated tumor killing in CRISPR screens. Regarding biomarker relevance, out of the 8 standardized biomarkers of response outcome and overall survival, the CHOL-Hub gene set achieved higher counts of the predictive score (AUC > 0.5) than TMB, T-cell clonality, and B-cell clonality (Figure 7C). Our results suggest that high expression levels of the CHOL-Hub genes are associated with worse outcomes to PD1, CTL4A, and PDL1 immunotherapies in various ICB datasets (Figure 7D). In addition, high expression levels of the hub genes predicted dysfunctional T-cell phenotypes but demonstrate no significant association with T-cell exclusion phenotypic markers (MDSC, CAF, and M2_TAM). However, the overall prioritization of the genes in the various immune-related datasets occurs in the order of HMGA2 > SNX15 > ATP2A1 > BET1 > PDCD10. Collectively, these data suggest that the high expression levels of the CHOL-Hub gene are associated with worse immune-oncological phenotypes and treatment responses, hence serving as an attractive target for therapy exploration.

### 3.6. Discovery of a Novel Small Molecule, HLC-018, via Scaffold-Hopping of Bioactive Compounds

**Figure 8:**
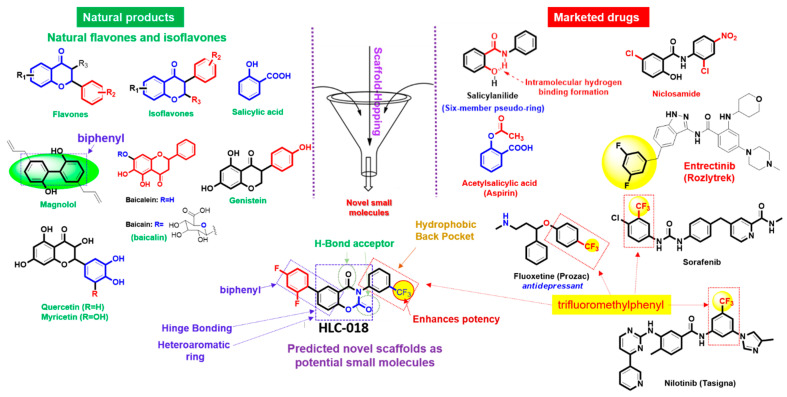
Scaffold-hopping of bioactive natural products and marketed drugs for the discovery of HLC-018. Scaffold-hopping of bioactive natural compounds (flavones and isoflavones), biphenyl, trifluoromethylphenyl, and niclosamide lead to the discovery of a novel small molecule, 6-(2,4-difluorophenyl)-3-(3-(trifluoromethyl)phenyl)-2H-benzo[e][1,3]oxazine-2,4(3H)-dione (HLC-018).

Scaffold-hopping of bioactive compounds is an important approach for novel drug design and development [45]. Biphenyl, flavones, and isoflavones are important natural product backbones and several bioactive compounds containing these backbones have been reported for a vast range of biological activities including anti-oxidative, anti-atherosclerosis, muscle relaxant, anti-microbial, anti-inflammatory, and anti-cancer effects [46,47]. Trifluoromethylphenyl is an important moiety that has been implicated in the activities of various drugs (Figure 8). A number of clinical drugs, e.g., nilotinib (a tyrosine kinase inhibitor with antineoplastic activity), fluoxetine (antidepressant, antiobsessional, anti-anxiety, and immunomodulating agent) and sorafenib (RAF/MEK/ERK inhibitor with anticancer activity) contain trifluoromethylphenyl as an important component responsible for their bioactivity. Niclosamide is a multipurpose compound with proven efficacy in the treatment of several diseases including oxidative stress, infection, metabolic disorders, inflammation and cancers [48,49]. In the present study, a scaffold-hopping (Figure 8) of these bioactive natural compounds (flavones and isoflavones), biphenyl, trifluoromethylphenyl, and niclosamide lead to the discovery of a novel multitarget small molecule; 6-(2,4-difluorophenyl)-3-(3-(trifluoromethyl)phenyl)-2H-benzo[e][1,3]oxazine-2,4(3H)-dione (HLC-018). Subsequently, we explored the potential of this compound as a therapeutic target against CHOL-hub genes through an in silico ligand-receptor interaction study.

### 3.7. Molecular Docking Reveals the Potential Druggability of CHOL-Hub Genes by HLC-018

**Figure 9:**
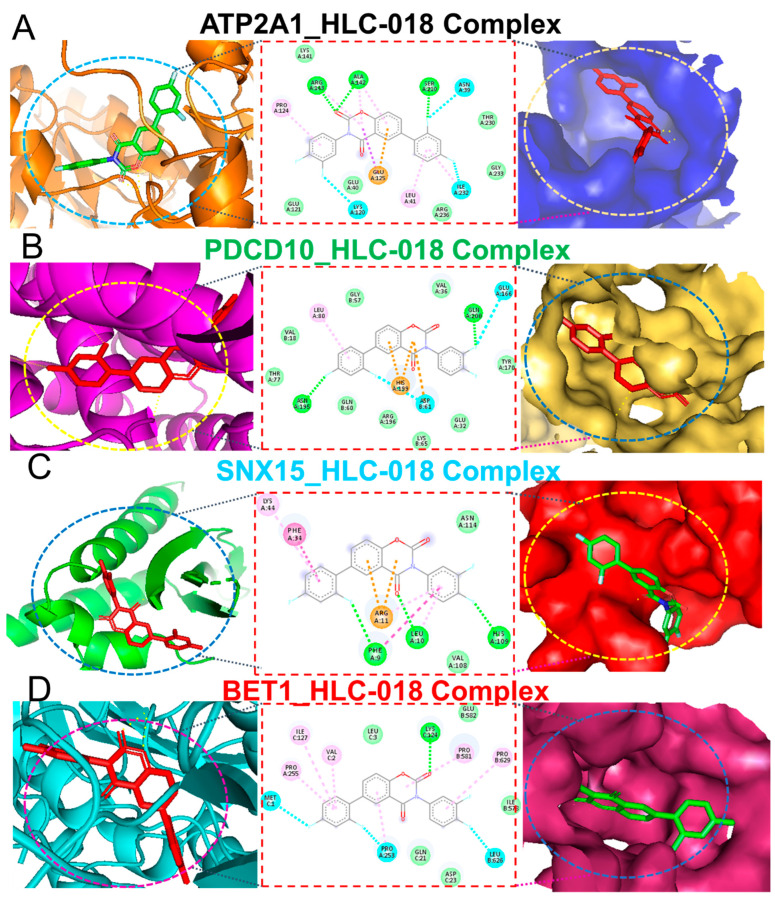
Ligand-receptor interaction profiles of HLC-018 in complex with (**A**) *ATP2A1*, (**B**) *PDCD10*, (**C**) *SNX15*, and (**D**) *BET1*. The left and middle panels respectively show two-(2D) and three-dimensional representations of ligand-receptor complexes, which reveal interacting amino acid residues and the type of interactions between the CHOL-hub genes and HLC-018. The right panels show the position of HLC-018 at the binding pocket flap of CHOL-hub genes.

Our molecular docking analysis revealed that HLC-018 exhibited high affinities for CHOL-hub genes with respective binding energies of −7.90, −10.40, −9.10, and −8.70 kcal/mol for *SNX15*, *ATP2A1*, *PDCD10*, and *BET1* (Table 3). HLC-018 bound to the binding pocket of CHOL-hub genes by several conventional H-bonds, halogen bonds, and multiple π-interactions (Figure 9). Furthermore, several van der Waals forces were found around the HLC-018 backbone with the respective amino acid residues of CHOL-hub gene binding pockets. Furthermore, ligand-receptor complexes were stabilized by various hydrophobic contacts. Altogether, the receptor-ligand interaction profile suggested that HLC-018 has high potential to target the CHOL-hub gene, having higher affinity for *PDCD10* and *ATP2A1* than for *SNX15* or *BET1*.

### 3.8. HLC-018 Demonstrated Specific Interactions with the Conserved Sequence of the AT-Hook DNA-Binding Motif of HMGA2

**Figure 10:**
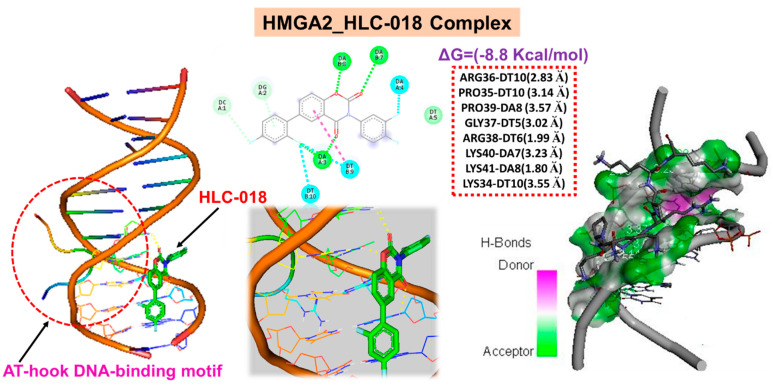
Molecular docking of HLC-018 with the unique Pro-Arg-Gly-Arg-Pro sequence of the AT-hook DNA-binding motif of HMGA2.

High-mobility group A2 (HMGA2) is an AT-hook DNA-binding motif-containing protein, identifiable by a conserved core sequence of Pro-Arg-Gly-Arg-Pro [50]. Our molecular docking study revealed a unique interaction of HLC-018 with the unique Pro-Arg-Gly-Arg-Pro sequence of the AT-hook DNA-binding motif of HMGA2 (Figure 10). We found that HLC-018 bound with hydrogen bonds to the PRO35_DT10-ARG36_DT10-GLY37_DT5-ARG38_DT6-PRO39_DA8 residues of HMGA2 with a relatively close proximity range of 1.99~3.55 Å and a binding affinity of −8.8 kcal/mol. No hydrophobic interactions were found.

## 4. Discussion

In recent years, great efforts have been put into studying the pathogenesis of CHOL, with much emphasis on the mechanisms of genetic and epigenetic alterations. However, our current knowledge of the genetic alterations and known molecular markers of CHOL is insufficient. Understanding the biology and pathogenesis of CHOL and its complex interactions with the TME could lead to better therapies and patient prognoses. The TME, a composite of various cells including stroma cells, infiltrating immune cells, and immunosuppressive cells, plays a crucial role in the initiation and progression of various human cancers [51].

In the present study, our attempt to explore novel genetic markers associated with the carcinogenesis of CHOL led to the identification of five genes (*SNX15*, *ATP2A1*, *PDCD10*, *BET1*, and *HMGA2*) as the most common deregulated overexpressed genes via integration of DEGs from relatively large-sample CHOL GEO datasets. We further explored the biological functions of CHOL-hub genes and found significant enrichment in several biological processes and pathways associated with stem cell angiogenesis, cell proliferation, and cancer development, while the interaction network revealed high genetic interactions with a number of onco-functional genes. In addition, we established associations between the CHOL-hub genes and tumor progression, metastasis, tumor immune and immunosuppressive cell infiltration, dysfunctional T-cell phenotypes, poor prognoses, and therapeutic resistance in CHOL. Therefore, it could plausibly be hypothesized that *SNX15*, *ATP2A1*, *PDCD10*, *BET1*, and *HMGA2* are major factors in CHOL and potential drivers of invasive immunophenotypes of CHOL. Results of the present study therefore add to the existing knowledge of the oncogenic roles of these genes, promote our understanding of the biology and carcinogenesis of CHOL, and provide new targets for risk stratification, molecular diagnoses, therapy exploration, and follow-up of CHOL.

Suggestive evidence from previous studies revealed that aggressive phenotypes and prognoses of cancers are largely dependent on tumor histology, stages, and metastasis. Interestingly, our analysis revealed that *SNX15*, *ATP2A1*, *PDCD10*, *BET1*, and *HMGA2* expressions were significantly associated with tumor stages and axillary lymph nodes metastasis of CHOL, and higher expression levels of CHOL-hub genes were significantly associated with higher risks and significantly worse prognoses (HR = 4.61, *p* = 0.044) of CHOL patients. In line with our observations, oncogenic roles of *PDCD10* were reported in various cancers including breast [52], bladder [53], prostate [54], ovarian [55], and several other cancers. A previous study also identified *ATP2A1* as an important driver of human papillomavirus (HPV)-associated oropharyngeal cancer [56]. The BET domain is regarded as a co-regulator of obesity, inflammation, and cancer [57]. Michalak et al. [58] identified BET1 as a malignancy-associated protein in human colorectal cancer, and inhibitors of BET1 were proposed for treating metastatic prostate cancer [59]. SNX15 is a regulator of intracellular protein trafficking consisting of endocytosis, endosomal sorting, and endosomal signaling and was associated with mammary adenocarcinoma metastases to the lungs [60].

HMGA2 is an AT-hook DNA-binding motif-containing protein, identifiable by a conserved core sequence of Pro-Arg-Gly-Arg-Pro [50]. It is a transcription factor that binds to the minor groove of the adenine-thymine (AT)-rich DNA sequence of several genes and alters the chromatin architecture with consequent modulation of their transcription [61]. As such, it influences various biological processes associated with cell proliferation and carcinogenesis. In agreement with our identification of HMGA2 as an important driver of CHOL carcinogenesis, overexpression of HMGA2 was reported in various cancers [62,63], and it was implicated in tumor metastasis, poor prognoses, and therapy failures in various cancer [64,65]. Thus, HMGA2 could serve as an important oncogenic molecule for exploration of targeted therapies.

Tumor microenvironment (TME), a composite of tumor cells, immune cells, stromal cells, cytokines, chemokines, and microvessels, has been proven to be crucial for the development of various tumors [39]. Although recent years have witnessed the emergence of novel treatment targets, medical therapy remains a compelling challenge in hepatobiliary malignancies [8,9,10]. The heterogenic complexity of CHOLs supported by the rich tumor microenvironment (TME) is a major contributor to high therapeutic failure [3]. Furthermore, dynamic regulatory mechanisms of interactions between the stromal and immune components of the TME in the progression of CHOL remain poorly understood. Results of the present study revealed that the CHOL-Hub genes are association with the infiltration levels of immune and immunosuppressive cells in cholangiocarcinoma. Tumor-infiltration of various immune and immunosuppressive cells vary with the host immune status and have latent prognostic value in various cancers. CAF, Treg, TAM, and MSDC are immunosuppressive cells that inhibit the function and abundance of cytotoxic lymphocytes leading to T-cell exclusion of the tumor and mediate invasive phenotypes [66]. Although, some tumors have abundant infiltration of immune cells, these immune cells are in a dysfunctional state and could not mediate any antitumor immune response, a condition known as T-cell allergy [41,51]. Interestingly, we found that high expression levels of the hub genes predicted dysfunctional T-cell phenotypes but demonstrate no significant association with T-cell exclusion phenotypic markers (MDSC, CAF, Treg, and TAM), suggesting that the CHOL hub genes are associated with invasive phenotypes of cholangiocarcinoma via dysfunctional T-cell phenotypes and not by T-cell exclusion mechanisms.

DNA methylation is an important epigenetic modulation in mammalian genomes, plays a crucial role in regulating gene expression, and may serve as a noninvasive biomarker for cancer diagnosis and prognosis [51,67]. It has been reported that DNA methylation induced genetic alterations in favor of carcinogenesis via the recruitment of the methyl moieties containing gene repressor proteins or by inhibiting the binding of the transcription factors to DNA [51,67]. Therefore, the differential methylation of CHOL-hub genes in cholangiocarcinoma when compared with the normal tissue strongly suggests that the CHOL-hub genes are involved in the epigenetic mechanism of the CHOL pathogenesis. In addition, the differential methylation of CHOL-hub genes predicted dysfunctional T-cell phenotypes and a high survival risk of the cohorts, suggesting that epigenetic modification of these genes does not only modulate the gene expression but also contributed to the immuno-invasive phenotypes and worse prognosis of cholangiocarcinoma.

Deciphering of genetic interactions is vital to understanding cellular and organismal responses to gene-level alterations in cancer [44]. Results of our GGI analysis found that CHOL-hub genes exhibited high genetic interactions with a number of onco-functional genes, particularly members of the GCKIII protein kinase family, which comprises MST3/STK24, SOK1/STK25, and MST4/STK26, and the SNARE proteins (GOSR1 and GOSR2). These are recognized as functional genes implicated in cell proliferation and the developments of various cancers [68,69]. Coherently, our biological functional and pathway analysis of CHOL-hub genes achieved enrichment of SNARE interactions, microRNAs in cancer, transcriptional misregulation, regulation of blood vessel endothelial cell proliferation involved in sprouting angiogenesis, regulation of stem cell proliferation, wound healing, spreading of cells, regulation of cell migration involved in sprouting angiogenesis, mitotic G_2_/M transition checkpoint, and the EMT. Altogether, our results suggested the CHOL-hub genes mediate the development and progression of CHOL via involvement in transcriptional misregulation, stem cell angiogenesis, cell proliferation, and cancer development.

Our in silico drug sensitivity analysis in various cancer cell lines suggested contrasting roles of CHOL-hub genes in potentiating therapeutic resistance. We found that *HMGA2* was strongly positively correlated, while *ATP2A1* was demonstrated to be strongly negatively correlated with IC_50_ values of all 30 CTRP small-molecule drugs, suggesting sharply contrasting roles of the former compared to the latter in modulating the sensitivity of cancer cell lines to therapy. Similarly, IC_50_ values of most of the small molecules from the GDSC database showed positive correlations with mRNA expression levels of *BET1* and *HMGA2*, suggesting their associations with therapeutic resistance, while *ATP2A1,* in stark contrast to *BET1* and *HMGA2*, demonstrated negative associations with IC_50_ values of the drugs. The potential mechanisms of the CHOL-hub genes’ effects on therapeutic responses require further investigation.

Nevertheless, we proposed that targeting CHOL-hub genes could become an ideal therapeutic approach for treating CHOL. To this end, we explored the potential of HLC-018, a novel small-molecule derivative using molecular docking of ligand-receptor complexes. Interestingly, our results revealed the potential druggability of CHOL-hub genes by HLC-018. To our delight, HLC-018 was well accommodated with high binding affinities to binding pockets of CHOL-hub genes. HLC-018 bound to the binding pockets of CHOL-hub genes by several conventional H-bonds, halogen bonds, and multiple π-interactions. Furthermore, several van der Waals forces were found around the HLC-018 backbone with respective amino acid residues of binding pockets of CHOL-hub genes. Furthermore, ligand-receptor complexes were stabilized by various hydrophobic contacts. Altogether, receptor-ligand interaction profiles suggested high potential of HLC-018 for targeting *PDCD10* and *ATP2A1* than for *SNX15* or *BET1*. More, importantly, we found that HLC-018 bound to the AT-hook DNA-binding motif of HMGA2 specifically at the conserved sequence of PRO35_DT10-ARG36_DT10-GLY37_DT5-ARG38_DT6-PRO39_DA8 all due to hydrogen bonds and with a high affinity of −8.8 kcal/mol. Compared to the binding affinity of this conserved region to some clinical drugs reported in the literature, such as with aspirin (−7.85 kcal/mol) and sulindac sulfide (−7.68 kcal/mol) [62], HLC-018 demonstrated a higher potential for binding to the AT-hook DNA-binding motif of *HMGA2* and consequently a higher potential for inhibiting the binding of the AT region of genes thereby affecting the transcriptional role of *HMGA2*. Altogether, our study provided insights into the immune-oncogenic phenotypes of CHOL and provided valuable information for ongoing experimental validation of targeted efficacy of HLC-018 in CHOL cell lines. Despite the advantages discussed above, the limitations of the present study merit discussion. This study represents an in silico and correlation analysis based on clinical data of cholangiocarcinoma patients and receptor-ligand interactions. Therefore, the reliability and clinical applicability of these findings required experimental validation. Furthermore, the full therapeutic potential of HLC-018 for targeting the CHOL-hub genes awaits experimental validation through in vitro and in vivo models.

## 5. Conclusions

In conclusion, the results of the present study suggest that the cholangiocarcinoma (CHOL)-hub genes (SNX15, ATP2A1, PDCD10, BET1, and HMGA2) can be used as biomarkers of tumor progression, metastasis, therapy outcome, and poor prognoses of CHOL. The CHOL-Hub genes potentially promote tumor progression by crosstalking with multiple oncogenic targets/pathways and potential drivers of invasive immunophenotypes of CHOL. To our delight, HLC-018, a novel benzamide-linked small molecule, demonstrated a high potential for targeting these genes and is currently under detailed experimental validation in our lab.

## Figures and Tables

**Table 1:** Characteristic of the cholangiocarcinoma (CHOL) datasets used for differentially expressed gene identification.

GEO Accession No.	Tumor Classification	Platform	CHOL	CONT	Total
GSE132305	Extrahepatic	GPL13667[HG-U219] Affymetrix Human Genome U219 array	182	38	220
GSE31370	Extrahepatic	GPL10558Illumina HumanHT-12 V4.0 expression beadchip	6	5	11
GSE38860	Intrahepatic	GPL8490; Illumina HumanMethylation27 BeadChip (HumanMethylation27_270596_v.1.2)	28	6	34
GSE45001	Intrahepatic	GPL14550; Agilent-028004 SurePrint G3 Human GE 8 × 60 K Microarray (Probe Name Version)	10	10	20
GSE32225	Intrahepatic	GPL8432; Illumina HumanRef-8 WG-DASL v3.0	149	6	155

CONT, control.

**Table 2:** Correlation of cholangiocarcinoma (CHOL)-hub gene mRNA expression levels and infiltration by tumor immune cells.

Symbol	Cell Type	Correlation	*p* Value	FDR	Entrez
ATP2A1	Monocyte	−0.32341	0.030233	0.090964	487
ATP2A1	Macrophage	−0.29581	0.048503	0.1206	487
ATP2A1	B cell	0.359449	0.015305	0.060663	487
BET1	Th1	0.298373	0.0465	0.281246	10,282
BET1	Macrophage	0.324999	0.029382	0.08435	10,282
HMGA2	Th17	−0.48051	0.000835	0.008929	8091
HMGA2	Monocyte	−0.37835	0.010386	0.047066	8091
HMGA2	Macrophage	−0.30952	0.038545	0.102486	8091
HMGA2	B cell	0.334685	0.024627	0.080284	8091
HMGA2	NKT	0.371549	0.011972	0.242193	8091
HMGA2	nTreg	0.426817	0.003457	0.019222	8091
HMGA2	CD8 naive	0.579818	2.99 × 10^−5^	0.002669	8091
PDCD10	MAIT	−0.43828	0.002601	0.05593	11,235
PDCD10	NK	−0.37253	0.011733	0.073089	11,235
PDCD10	Monocyte	−0.33388	0.024995	0.080076	11,235
PDCD10	B cell	0.29797	0.04681	0.121704	11,235
PDCD10	CD4_T	0.309234	0.038735	0.210845	11,235
PDCD10	DC	0.315952	0.034487	0.217638	11,235
PDCD10	iTreg	0.370646	0.012197	0.237308	11,235
PDCD10	nTreg	0.39415	0.007383	0.030899	11,235
PDCD10	Tr1	0.510549	0.000339	0.008799	11,235
SNX15	Macrophage	−0.31268	0.036506	0.098523	29,907
	**Copy Number Alterations**				
BET1	Gamma_delta	−0.43421	0.008145	0.827591	10,282
BET1	MAIT	0.402937	0.014826	0.546837	10,282
BET1	Macrophage	0.353956	0.034184	0.678796	10,282
SNX15	Tr1	0.344195	0.039824	0.445423	29,907
	**Methylation**				
BET1	CD4_naive	−0.41012	0.012983	0.362536	10,282
BET1	Gamma_delta	0.38743	0.019567	0.844713	10,282
ATP2A1	Neutrophil	−0.37255	0.025246	0.166953	487
HMGA2	Tr1	−0.37059	0.026084	0.156054	8091
ATP2A1	nTreg	0.333934	0.046542	0.999274	487

**Table 3:** Docking profile of HLC-018 with cholangiocarcinoma (CHOL)-hub genes.

	HLC-018/CHOL-Hub Gene Complexes			
Interaction	*SNX15*	*ATP2A1*	*PDCD10*	*BET1*
ΔG = (kcal/mol)	−7.90	−10.40	−9.10	−8.70
Conventional H-bonds	HIS109 (2.04) LEU10 (2.05) PHE9 (2.62)	ARG143 (2.38) ALA142 (3.01) SER210 (2.22)	GLN200 (2.66) ASN195 (2.51)	LYS124 (2.86)
Halogen bonds		ASN39	GLU166, ASP61	MET1, PRO253, LEU626
π-alkyl	LYS44	PRO124, LEU41	LEU80	ILE127, PRO255, VAL2, PRO581, PRO629
π-π stacked	PHE34, PHE9			
π-cation	ARG11	GLU125	HIS199	
π-anion			ASP61	
van der Waals forces	VAL108, ASN114	GLU121, ARG236, GLU40, LYS141, THR230, GLY233	GLY57, VAL18, THR77, GLN60, ARG196, GLU32, TYR170, VAL36, LYS65	GLN21, ASP23, LEU3, GLU582, ILE578
Hydrophobic interactions (Å)	PHE9A (3.52) ARG11A (3.96) PHE34A (3.45) LYS44A (3.57)	LEU41A (3.74) PRO124A (3.82) ALA142A (3.50) ILE232A (3.77)	GLU32A (3.99) GLN60B (3.66) ASP61B (3.84) LEU80A (3.63) ASN195A (3.68)	VAL2C (3.93) ILE127C (3.66) PRO253A (3.62) PRO629B (3.67)

## Data Availability

The raw data supporting the conclusions of this article will be made available by the authors, without undue reservation, to any qualified researcher.

## Supplementary Materials

The following are available online at https://www.mdpi.com/article/10.3390/cells10112873/s1, Table S1: Multivariable and HR analysis of the CHOL hub genes in cholangiocarcinoma. Table S2: Coexpression interactions of CHOL-hub genes with the expression levels of the onco-functional genes. Figure S1: Heatmap of the expression levels of the CHOL-Hub genes based on single-cell RNA-sequencing dataset.
