# Supplementary material for: Transcriptomic-Based Identification of the Immuno-Oncogenic Signature of Cholangiocarcinoma for HLC-018 Multi-Target Therapy Exploration
Source: Cells. 2021 Oct 25;10(11):2873. doi: 10.3390/cells10112873 (PMC8616156; doi:10.3390/cells10112873)
Supplement: Supplementary file 1 [file cells-10-02873-s001.zip › cells-1413200-supplementary.pdf]

Table S1: Multivariable and HR analysis of the CHOL hub genes in cholangiocarcinoma.

HMGA2 in CHOL (n=36):
Model: Surv(OS, EVENT) ~ `HMGA2` + Age + Stage + Purity + Race + Gender
36 patients with 18 dying (0 missing obs.)
coef HR se(coef) 95%CI_l 95%CI_u z p signif
HMGA2 0.649 1.914 0.274 1.119 3.274 2.370 0.018 *
Age 0.013 1.013 0.023 0.969 1.060 0.574 0.566
Stage2 1.150 3.157 0.798 0.661 15.073 1.441 0.150
Stage3 -15.427 0.000 8166.087 0.000 Inf -0.002 0.998
Stage4 1.273 3.572 0.756 0.811 15.729 1.683 0.092 .
RaceBlack 1.596 4.933 1.653 0.193 126.035 0.965 0.334
RaceWhite 0.691 1.996 1.219 0.183 21.770 0.567 0.571
Gendermale 0.386 1.472 0.617 0.439 4.932 0.626 0.531
Rsquare = 0.323 (max possible = 9.46e-01)
Likelihood ratio test p = 1.21e-01
Wald test p = 2.06e-01
Score (logrank) test p = 5.06e-02
PDCD10 in CHOL (n=36):
Model: Surv(OS, EVENT) ~ `PDCD10` + Age + Stage + Purity + Race + Gender
36 patients with 18 dying (0 missing obs.)
coef HR se(coef) 95%CI_l 95%CI_u z p signif
PDCD10 -0.501 0.606 0.513 0.222 1.656 -0.977 0.329
Age 0.014 1.014 0.023 0.968 1.061 0.578 0.563
Stage2 0.536 1.709 0.712 0.423 6.897 0.753 0.452
Stage3 -16.068 0.000 7001.668 0.000 Inf -0.002 0.998
Stage4 1.036 2.817 0.704 0.709 11.192 1.471 0.141
Purity 1.796 6.028 1.629 0.248 146.726 1.103 0.270
RaceBlack -0.923 0.397 1.641 0.016 9.906 -0.562 0.574
RaceWhite -1.349 0.259 0.956 0.040 1.689 -1.411 0.158
Gendermale 0.175 1.191 0.565 0.394 3.604 0.310 0.757
Rsquare = 0.232 (max possible = 9.46e-01)
Likelihood ratio test p = 3.91e-01
Wald test p = 5.84e-01
Score (logrank) test p = 4.02e-01
BET1 in CHOL (n=36):
Model: Surv(OS, EVENT) ~ `BET1` + Age + Stage + Purity + Race + Gender
36 patients with 18 dying (0 missing obs.)
coef HR se(coef) 95%CI_l 95%CI_u z p signif
BET1 0.453 1.572 0.700 0.398 6.206 0.646 0.518
Age 0.019 1.019 0.021 0.977 1.063 0.887 0.375
Stage2 0.773 2.166 0.691 0.559 8.393 1.118 0.264
Stage3 -15.002 0.000 6962.378 0.000 Inf -0.002 0.998

Stage4 0.797 2.218 0.660 0.609 8.082 1.207 0.227
Purity 2.566 13.013 1.787 0.392 432.380 1.436 0.151
RaceBlack -0.355 0.701 1.480 0.039 12.748 -0.240 0.810
RaceWhite -1.114 0.328 0.896 0.057 1.900 -1.244 0.214
Gendermale 0.305 1.357 0.572 0.442 4.164 0.533 0.594
Rsquare = 0.22 (max possible = 9.46e-01)
Likelihood ratio test p = 4.42e-01
Wald test p = 6.34e-01
Score (logrank) test p = 4.65e-01
Model: Surv(OS, EVENT) ~ \`ATP2A1\` + Age + Stage + Purity + Race + Gender
36 patients with 18 dying (0 missing obs.)
coef HR se(coef) 95%CI_l 95%CI_u z p signif
ATP2A1 0.034 1.035 0.264 0.617 1.734 0.129 0.897
Age 0.017 1.018 0.022 0.975 1.062 0.795 0.427
Stage2 0.651 1.917 0.677 0.508 7.230 0.960 0.337
Stage3 -15.521 0.000 6948.172 0.000 Inf -0.002 0.998
Stage4 0.845 2.329 0.691 0.602 9.018 1.224 0.221
Purity 1.996 7.360 1.588 0.327 165.572 1.257 0.209
RaceBlack -0.358 0.699 1.494 0.037 13.064 -0.240 0.811
RaceWhite -1.097 0.334 0.911 0.056 1.993 -1.203 0.229
Gendermale 0.263 1.300 0.568 0.427 3.962 0.462 0.644
Rsquare = 0.212 (max possible = 9.46e-01)
Likelihood ratio test p = 4.79e-01
Wald test p = 6.53e-01
Score (logrank) test p = 4.87e-01
SNX15 in CHOL (n=36):
Model: Surv(OS, EVENT) ~ \`SNX15\` + Age + Stage + Purity + Race + Gender
36 patients with 18 dying (0 missing obs.)
coef HR se(coef) 95%CI_l 95%CI_u z p signif
SNX15 -0.634 0.530 0.485 0.205 1.372 -1.307 0.191
Age 0.025 1.026 0.023 0.981 1.073 1.109 0.267
Stage2 0.782 2.186 0.654 0.606 7.882 1.196 0.232
Stage3 -15.163 0.000 7018.953 0.000 Inf -0.002 0.998
Stage4 0.679 1.973 0.694 0.506 7.692 0.979 0.328
Purity 2.641 14.022 1.674 0.527 372.952 1.578 0.115
RaceBlack 0.164 1.178 1.519 0.060 23.118 0.108 0.914
RaceWhite -1.137 0.321 0.899 0.055 1.869 -1.265 0.206
Gendermale 0.345 1.412 0.564 0.467 4.268 0.612 0.541
Rsquare = 0.249 (max possible = 9.46e-01)
Likelihood ratio test p = 3.26e-01
Wald test p = 5.25e-01
Score (logrank) test p = 3.58e-01

Table S2: Coexpression interactions of CHOL-hub genes with the expression levels of the onco-functional genes.

Gene 1	Gene 2	Weight	Network group	Network
SLN	ATP2A1	0.014872	Co-expression	Mallon-McKay-2013
SEC22B	BET1	0.010436	Co-expression	Roth-Zlotnik-2006
SLN	ATP2A1	0.008375	Co-expression	Roth-Zlotnik-2006
GOSR1	BET1	0.010026	Co-expression	Roth-Zlotnik-2006
ASF1A	PDCD10	0.010139	Co-expression	Roth-Zlotnik-2006
STK24	PDCD10	0.006637	Co-expression	Ramaswamy-Golub-2001
GOSR1	STX5	0.012954	Co-expression	Ramaswamy-Golub-2001
STX7	GOSR2	0.012267	Co-expression	Ramaswamy-Golub-2001
SEC22B	BET1	0.010875	Co-expression	Innocenti-Brown-2011
STK26	PDCD10	0.006019	Co-expression	Innocenti-Brown-2011
HMGA1	PDCD10	0.014497	Co-expression	Alizadeh-Staudt-2000
NOTCH1	STK24	0.020278	Co-expression	Alizadeh-Staudt-2000
E4F1	STK24	0.019253	Co-expression	Alizadeh-Staudt-2000
SLN	ATP2A1	0.006273	Co-expression	Dobbin-Giordano-2005
PDGFRB	NOTCH1	0.007634	Co-expression	Dobbin-Giordano-2005
UBN1	E4F1	0.019025	Co-expression	Dobbin-Giordano-2005
GOSR1	SEC22B	0.017188	Co-expression	Rieger-Chu-2004
PDGFRB	NAPA	0.018297	Co-expression	Rieger-Chu-2004
ASF1A	PDCD10	0.016075	Co-expression	Rieger-Chu-2004
SEC22B	BET1	0.012847	Co-expression	Bild-Nevins-2006 B
STX7	PDCD10	0.020381	Co-expression	Bild-Nevins-2006 B
ASF1A	STK24	0.007647	Co-expression	Boldrick-Relman-2002
NAPA	STX5	0.013415	Co-expression	Jiang-de Kok-2017
STX7	BET1	0.013188	Co-expression	Jiang-de Kok-2017
VTI1A	YKT6	0.029964	Co-expression	Jiang-de Kok-2017
PDCD10	BET1	0.020505	Co-expression	Perou-Botstein-2000
SEC22B	BET1	0.012501	Co-expression	Chen-Brown-2002
HMGA1	PDCD10	0.00789	Co-expression	Chen-Brown-2002
UBN1	HMGA1	0.009452	Co-expression	Chen-Brown-2002
HMGA1	HMGA2	0.007358	Co-expression	Wang-Cheung-2015
YKT6	GOSR1	0.0186	Co-expression	Wang-Cheung-2015
ASF1A	SEC22B	0.013787	Co-expression	Wang-Cheung-2015
ASF1A	STK26	0.011162	Co-expression	Wang-Cheung-2015
SLN	ATP2A1	0.003798	Co-expression	Wu-Garvey-2007
NAPA	STK25	0.007763	Co-expression	Wu-Garvey-2007
E4F1	STK24	0.017731	Co-expression	Rosenwald-Staudt-2001
NAPA	GOSR1	0.016825	Co-expression	Ross-Perou-2001
HMGA1	PDCD10	0.013197	Co-localization	Johnson-Shoemaker-2003
GOSR1	PDCD10	0.001533	Genetic Interactions	Lin-Smith-2010
GOSR1	STX5	0.001144	Genetic Interactions	Lin-Smith-2010
HMGA1	BET1	0.008165	Genetic Interactions	Lin-Smith-2010
YKT6	STK25	0.001702	Genetic Interactions	Lin-Smith-2010
PDGFRB	NOTCH1	0.001736	Genetic Interactions	Lin-Smith-2010
VTI1A	STK25	0.000479	Genetic Interactions	Lin-Smith-2010
VTI1A	SLN	0.000811	Genetic Interactions	Lin-Smith-2010
HIRA	STX7	0.001032	Genetic Interactions	Lin-Smith-2010
STX5	BET1	0.35807	Pathway	Wu-Stein-2010
SEC22B	BET1	0.306441	Pathway	Wu-Stein-2010
SEC22B	STX5	0.132749	Pathway	Wu-Stein-2010
GOSR1	STX5	0.370097	Pathway	Wu-Stein-2010
YKT6	STX5	0.127591	Pathway	Wu-Stein-2010
YKT6	GOSR1	0.304427	Pathway	Wu-Stein-2010
STX7	YKT6	0.116059	Pathway	Wu-Stein-2010
PDGFRB	SNX15	0.305519	Pathway	Wu-Stein-2010
VTI1A	YKT6	0.097672	Pathway	Wu-Stein-2010
VTI1A	STX7	0.108009	Pathway	Wu-Stein-2010
HMGA1	HMGA2	0.051771	Physical Interactions	IREF-reactome
NOTCH1	ATP2A1	0.220696	Physical Interactions	IREF-reactome
ASF1A	HMGA2	0.133488	Physical Interactions	IREF-reactome
ASF1A	HMGA1	0.051771	Physical Interactions	IREF-reactome
UBN1	HMGA2	0.133488	Physical Interactions	IREF-reactome

UBN1	HMGA1	0.051771	Physical Interactions	IREF-reactome
UBN1	ASF1A	0.133488	Physical Interactions	IREF-reactome
HIRA	HMGA2	0.133488	Physical Interactions	IREF-reactome
HIRA	HMGA1	0.051771	Physical Interactions	IREF-reactome
HIRA	ASF1A	0.133488	Physical Interactions	IREF-reactome
HIRA	UBN1	0.133488	Physical Interactions	IREF-reactome
HMGA1	HMGA2	0.051771	Physical Interactions	Vastrik-Stein-2007
NOTCH1	ATP2A1	0.220696	Physical Interactions	Vastrik-Stein-2007
ASF1A	HMGA2	0.133488	Physical Interactions	Vastrik-Stein-2007
ASF1A	HMGA1	0.051771	Physical Interactions	Vastrik-Stein-2007
UBN1	HMGA2	0.133488	Physical Interactions	Vastrik-Stein-2007
UBN1	HMGA1	0.051771	Physical Interactions	Vastrik-Stein-2007
UBN1	ASF1A	0.133488	Physical Interactions	Vastrik-Stein-2007
HIRA	HMGA2	0.133488	Physical Interactions	Vastrik-Stein-2007
HIRA	HMGA1	0.051771	Physical Interactions	Vastrik-Stein-2007
HIRA	ASF1A	0.133488	Physical Interactions	Vastrik-Stein-2007
HIRA	UBN1	0.133488	Physical Interactions	Vastrik-Stein-2007
STX5	BET1	0.254403	Physical Interactions	Liu-Varjosalo-2018
SEC22B	BET1	0.254403	Physical Interactions	Liu-Varjosalo-2018
GOSR1	BET1	0.254403	Physical Interactions	Liu-Varjosalo-2018
GOSR2	BET1	0.254403	Physical Interactions	Liu-Varjosalo-2018
YKT6	BET1	0.254403	Physical Interactions	Liu-Varjosalo-2018
NAPA	BET1	0.254403	Physical Interactions	Liu-Varjosalo-2018
STX7	BET1	0.254403	Physical Interactions	Liu-Varjosalo-2018
VTI1A	BET1	0.254403	Physical Interactions	Liu-Varjosalo-2018
STK24	PDCD10	0.054817	Physical Interactions	Kristensen-Foster-2012
UBN1	ASF1A	0.143376	Physical Interactions	Hein-Mann-2015
HIRA	ASF1A	0.207353	Physical Interactions	Hein-Mann-2015
STK25	PDCD10	0.32447	Physical Interactions	IREF-quickgo
STK24	PDCD10	0.241933	Physical Interactions	IREF-quickgo
STK26	PDCD10	0.241933	Physical Interactions	IREF-quickgo
E4F1	HMGA2	0.681855	Physical Interactions	IREF-quickgo
CCM2	PDCD10	0.301249	Physical Interactions	IREF-quickgo
STK25	PDCD10	0.605	Physical Interactions	IREF-dip
SEC22B	STX5	0.707107	Physical Interactions	IREF-dip
STK24	PDCD10	0.252697	Physical Interactions	IREF-dip
STK26	PDCD10	0.266692	Physical Interactions	IREF-dip
CCM2	PDCD10	0.242182	Physical Interactions	IREF-dip
HIRA	ASF1A	0.353542	Physical Interactions	IREF-dip
STK25	PDCD10	0.232021	Physical Interactions	Wan-Emili-2015
STK24	PDCD10	0.159031	Physical Interactions	Wan-Emili-2015
NAPA	BET1	0.240514	Physical Interactions	Wan-Emili-2015
STK26	PDCD10	0.23922	Physical Interactions	Wan-Emili-2015
STX7	BET1	0.179565	Physical Interactions	Wan-Emili-2015
NAPA	BET1	0.192245	Physical Interactions	Hubel-Pichlmair-2019
NAPA	STX5	0.084995	Physical Interactions	Hubel-Pichlmair-2019
NAPA	SEC22B	0.076087	Physical Interactions	Hubel-Pichlmair-2019
NAPA	GOSR1	0.100766	Physical Interactions	Hubel-Pichlmair-2019
NAPA	GOSR2	0.122139	Physical Interactions	Hubel-Pichlmair-2019
STX7	NAPA	0.122139	Physical Interactions	Hubel-Pichlmair-2019
VTI1A	NAPA	0.122139	Physical Interactions	Hubel-Pichlmair-2019
STX5	BET1	0.124252	Physical Interactions	BIOGRID-SMALL-SCALE-STUDIES
SEC22B	STX5	0.102422	Physical Interactions	BIOGRID-SMALL-SCALE-STUDIES
SLN	ATP2A1	0.431282	Physical Interactions	BIOGRID-SMALL-SCALE-STUDIES
GOSR1	BET1	0.23793	Physical Interactions	BIOGRID-SMALL-SCALE-STUDIES
GOSR1	STX5	0.06346	Physical Interactions	BIOGRID-SMALL-SCALE-STUDIES
GOSR2	STX5	0.236711	Physical Interactions	BIOGRID-SMALL-SCALE-STUDIES
YKT6	STX5	0.091622	Physical Interactions	BIOGRID-SMALL-SCALE-STUDIES
YKT6	GOSR1	0.175447	Physical Interactions	BIOGRID-SMALL-SCALE-STUDIES
NAPA	STX5	0.069058	Physical Interactions	BIOGRID-SMALL-SCALE-STUDIES
STX7	NAPA	0.095781	Physical Interactions	BIOGRID-SMALL-SCALE-STUDIES
E4F1	HMGA2	0.147005	Physical Interactions	BIOGRID-SMALL-SCALE-STUDIES
UBN1	ASF1A	0.021237	Physical Interactions	BIOGRID-SMALL-SCALE-STUDIES

VTI1A	STX7	0.150975	Physical Interactions	BIOGRID-SMALL-SCALE-STUDIES
HIRA	ASF1A	0.011352	Physical Interactions	BIOGRID-SMALL-SCALE-STUDIES
HIRA	UBN1	0.019622	Physical Interactions	BIOGRID-SMALL-SCALE-STUDIES
STK25	PDCD10	0.040174	Physical Interactions	Huttlin-Harper-2017
SEC22B	BET1	0.093082	Physical Interactions	Huttlin-Harper-2017
SEC22B	STX5	0.026547	Physical Interactions	Huttlin-Harper-2017
STK24	PDCD10	0.111386	Physical Interactions	Huttlin-Harper-2017
STK24	STK25	0.09435	Physical Interactions	Huttlin-Harper-2017
GOSR1	SEC22B	0.048973	Physical Interactions	Huttlin-Harper-2017
GOSR2	SEC22B	0.079433	Physical Interactions	Huttlin-Harper-2017
NAPA	SEC22B	0.081685	Physical Interactions	Huttlin-Harper-2017
STK26	PDCD10	0.067387	Physical Interactions	Huttlin-Harper-2017
STK26	STK25	0.057081	Physical Interactions	Huttlin-Harper-2017
STX7	STX5	0.016818	Physical Interactions	Huttlin-Harper-2017
STX7	SEC22B	0.017736	Physical Interactions	Huttlin-Harper-2017
STX7	GOSR1	0.031025	Physical Interactions	Huttlin-Harper-2017
STX7	GOSR2	0.050322	Physical Interactions	Huttlin-Harper-2017
STX7	YKT6	0.033622	Physical Interactions	Huttlin-Harper-2017
STX7	NAPA	0.051748	Physical Interactions	Huttlin-Harper-2017
CCM2	STK26	0.126739	Physical Interactions	Huttlin-Harper-2017
UBN1	ASF1A	0.198839	Physical Interactions	Huttlin-Harper-2017
VTI1A	STX7	0.066226	Physical Interactions	Huttlin-Harper-2017
HIRA	ASF1A	0.059135	Physical Interactions	Huttlin-Harper-2017
STK25	PDCD10	0.126071	Physical Interactions	Varjosalo-Superti-Furga-2013
STK24	PDCD10	0.13356	Physical Interactions	Varjosalo-Superti-Furga-2013
STK24	STK25	0.020128	Physical Interactions	Varjosalo-Superti-Furga-2013
STK26	PDCD10	0.149826	Physical Interactions	Varjosalo-Superti-Furga-2013
STK26	STK25	0.02258	Physical Interactions	Varjosalo-Superti-Furga-2013
STK26	STK24	0.023921	Physical Interactions	Varjosalo-Superti-Furga-2013
GOSR1	SEC22B	0.55472	Physical Interactions	Behrends-Harper-2010
STK25	PDCD10	0.352672	Physical Interactions	IREF-mint
STK24	PDCD10	0.441918	Physical Interactions	IREF-mint
CCM2	PDCD10	0.68125	Physical Interactions	IREF-mint
SLN	ATP2A1	1	Physical Interactions	IREF-corum
STK24	PDCD10	0.210249	Physical Interactions	Ewing-Figeys-2007
STK24	STK25	0.287009	Physical Interactions	Ewing-Figeys-2007
HIRA	ASF1A	0.242786	Physical Interactions	IREF-bind-translation
HIRA	ASF1A	0.26624	Physical Interactions	IREF-bind
STK25	PDCD10	0.108559	Physical Interactions	Huttlin-Gygi-2015
STK24	STK25	0.090932	Physical Interactions	Huttlin-Gygi-2015
STK26	STK25	0.07728	Physical Interactions	Huttlin-Gygi-2015
STX7	STX5	0.022267	Physical Interactions	Huttlin-Gygi-2015
STX7	GOSR1	0.056974	Physical Interactions	Huttlin-Gygi-2015
STX7	GOSR2	0.089229	Physical Interactions	Huttlin-Gygi-2015
STX7	YKT6	0.048129	Physical Interactions	Huttlin-Gygi-2015
STX7	NAPA	0.049728	Physical Interactions	Huttlin-Gygi-2015
VTI1A	STX7	0.089229	Physical Interactions	Huttlin-Gygi-2015
STK25	PDCD10	0.707107	Physical Interactions	Rual-Vidal-2005
STK24	PDCD10	0.707107	Physical Interactions	Rual-Vidal-2005
GOSR2	STX5	0.842676	Physical Interactions	Rual-Vidal-2005
NAPA	STX5	0.244919	Physical Interactions	Rual-Vidal-2005
NAPA	GOSR1	0.540949	Physical Interactions	Rual-Vidal-2005
STX7	NAPA	0.275204	Physical Interactions	Rual-Vidal-2005
STK25	PDCD10	0.532672	Physical Interactions	IREF-spike
STK24	PDCD10	0.257685	Physical Interactions	IREF-spike
STK24	STK25	0.333869	Physical Interactions	IREF-spike
GOSR2	STX5	0.648809	Physical Interactions	IREF-spike
NAPA	STX5	0.137267	Physical Interactions	IREF-spike
NAPA	GOSR1	0.176752	Physical Interactions	IREF-spike
STX7	NAPA	0.268955	Physical Interactions	IREF-spike
STK25	PDCD10	0.248325	Physical Interactions	IREF-huri
STK24	PDCD10	0.232181	Physical Interactions	IREF-huri
GOSR2	BET1	0.020496	Physical Interactions	IREF-huri

GOSR2	STX5	0.02856	Physical Interactions	IREF-huri
STK26	PDCD10	0.422518	Physical Interactions	IREF-huri
SLN	ATP2A1	0.17453	Physical Interactions	IREF-biogrid
YKT6	STX5	0.020193	Physical Interactions	IREF-biogrid
NAPA	STX5	0.021999	Physical Interactions	IREF-biogrid
STX7	STX5	0.013185	Physical Interactions	IREF-biogrid
STX7	YKT6	0.014192	Physical Interactions	IREF-biogrid
STX7	NAPA	0.015461	Physical Interactions	IREF-biogrid
HIRA	ASF1A	0.018834	Physical Interactions	IREF-biogrid
SEC22B	STX5	0.012484	Predicted	I2D-BioGRID-Yeast2Human
GOSR1	STX5	0.022172	Predicted	I2D-BioGRID-Yeast2Human
GOSR1	SEC22B	0.009463	Predicted	I2D-BioGRID-Yeast2Human
YKT6	STX5	0.040125	Predicted	I2D-BioGRID-Yeast2Human
YKT6	SEC22B	0.017126	Predicted	I2D-BioGRID-Yeast2Human
YKT6	GOSR1	0.030415	Predicted	I2D-BioGRID-Yeast2Human
NAPA	STX5	0.038488	Predicted	I2D-BioGRID-Yeast2Human
NAPA	SEC22B	0.016427	Predicted	I2D-BioGRID-Yeast2Human
NAPA	GOSR1	0.029174	Predicted	I2D-BioGRID-Yeast2Human
NAPA	YKT6	0.052797	Predicted	I2D-BioGRID-Yeast2Human
ASF1A	SEC22B	0.006524	Predicted	I2D-BioGRID-Yeast2Human
SEC22B	STX5	0.207164	Predicted	I2D-Li-Vidal-2004-interolog-Worm2Human
GOSR1	STX5	0.22891	Predicted	I2D-Li-Vidal-2004-interolog-Worm2Human
YKT6	STX5	0.22891	Predicted	I2D-Li-Vidal-2004-interolog-Worm2Human
YKT6	GOSR1	0.378699	Predicted	I2D-Li-Vidal-2004-interolog-Worm2Human
NAPA	GOSR1	0.260772	Predicted	I2D-Li-Vidal-2004-interolog-Worm2Human
VTI1A	STX5	0.176071	Predicted	I2D-Li-Vidal-2004-interolog-Worm2Human
VTI1A	YKT6	0.291284	Predicted	I2D-Li-Vidal-2004-interolog-Worm2Human
VTI1A	NAPA	0.200579	Predicted	I2D-Li-Vidal-2004-interolog-Worm2Human
STK24	STK25	0.030377	Predicted	Wu-Stein-2010
SLN	ATP2A1	0.328174	Predicted	Wu-Stein-2010
GOSR1	BET1	0.209187	Predicted	Wu-Stein-2010
GOSR1	SEC22B	0.279051	Predicted	Wu-Stein-2010
GOSR2	BET1	0.323555	Predicted	Wu-Stein-2010
GOSR2	STX5	0.138977	Predicted	Wu-Stein-2010
GOSR2	SEC22B	0.431616	Predicted	Wu-Stein-2010
GOSR2	GOSR1	0.132952	Predicted	Wu-Stein-2010
NAPA	STX5	0.058306	Predicted	Wu-Stein-2010
NAPA	GOSR1	0.055778	Predicted	Wu-Stein-2010
NAPA	GOSR2	0.086274	Predicted	Wu-Stein-2010
STX7	NAPA	0.078878	Predicted	Wu-Stein-2010
YKT6	BET1	0.574467	Predicted	I2D-BioGRID-Mouse2Human
YKT6	STX5	0.574467	Predicted	I2D-BioGRID-Mouse2Human
YKT6	GOSR1	0.574467	Predicted	I2D-BioGRID-Mouse2Human
VTI1A	STX7	0.221373	Predicted	I2D-BioGRID-Mouse2Human
SEC22B	STX5	0.297531	Predicted	I2D-Yu-Vidal-2008-GoldStd-Yeast2Human
GOSR1	STX5	0.519377	Predicted	I2D-Yu-Vidal-2008-GoldStd-Yeast2Human
YKT6	STX5	0.519377	Predicted	I2D-Yu-Vidal-2008-GoldStd-Yeast2Human
NAPA	SEC22B	0.374137	Predicted	I2D-Yu-Vidal-2008-GoldStd-Yeast2Human
STX5	BET1	0.054623	Predicted	I2D-BioGRID-Fly2Human
SEC22B	BET1	0.054623	Predicted	I2D-BioGRID-Fly2Human
SEC22B	STX5	0.052696	Predicted	I2D-BioGRID-Fly2Human
GOSR1	BET1	0.05662	Predicted	I2D-BioGRID-Fly2Human
GOSR1	STX5	0.054623	Predicted	I2D-BioGRID-Fly2Human
GOSR1	SEC22B	0.054623	Predicted	I2D-BioGRID-Fly2Human
GOSR2	BET1	0.054623	Predicted	I2D-BioGRID-Fly2Human
GOSR2	STX5	0.052696	Predicted	I2D-BioGRID-Fly2Human
GOSR2	SEC22B	0.052696	Predicted	I2D-BioGRID-Fly2Human
GOSR2	GOSR1	0.054623	Predicted	I2D-BioGRID-Fly2Human
NOTCH1	ATP2A1	0.008497	Predicted	I2D-BioGRID-Fly2Human
YKT6	BET1	0.05662	Predicted	I2D-BioGRID-Fly2Human
YKT6	STX5	0.054623	Predicted	I2D-BioGRID-Fly2Human
YKT6	SEC22B	0.054623	Predicted	I2D-BioGRID-Fly2Human
YKT6	GOSR1	0.05662	Predicted	I2D-BioGRID-Fly2Human

YKT6	GOSR2	0.054623	Predicted	I2D-BioGRID-Fly2Human
NAPA	BET1	0.050637	Predicted	I2D-BioGRID-Fly2Human
NAPA	STX5	0.04885	Predicted	I2D-BioGRID-Fly2Human
NAPA	SEC22B	0.04885	Predicted	I2D-BioGRID-Fly2Human
NAPA	GOSR1	0.050637	Predicted	I2D-BioGRID-Fly2Human
NAPA	GOSR2	0.04885	Predicted	I2D-BioGRID-Fly2Human
NAPA	YKT6	0.050637	Predicted	I2D-BioGRID-Fly2Human
STX7	BET1	0.042921	Predicted	I2D-BioGRID-Fly2Human
STX7	STX5	0.041407	Predicted	I2D-BioGRID-Fly2Human
STX7	SEC22B	0.041407	Predicted	I2D-BioGRID-Fly2Human
STX7	GOSR1	0.042921	Predicted	I2D-BioGRID-Fly2Human
STX7	GOSR2	0.041407	Predicted	I2D-BioGRID-Fly2Human
STX7	YKT6	0.042921	Predicted	I2D-BioGRID-Fly2Human
STX7	NAPA	0.038385	Predicted	I2D-BioGRID-Fly2Human
VTI1A	BET1	0.05672	Predicted	I2D-BioGRID-Fly2Human
VTI1A	STX5	0.054719	Predicted	I2D-BioGRID-Fly2Human
VTI1A	SEC22B	0.054719	Predicted	I2D-BioGRID-Fly2Human
VTI1A	GOSR1	0.05672	Predicted	I2D-BioGRID-Fly2Human
VTI1A	GOSR2	0.054719	Predicted	I2D-BioGRID-Fly2Human
VTI1A	YKT6	0.05672	Predicted	I2D-BioGRID-Fly2Human
VTI1A	NAPA	0.050726	Predicted	I2D-BioGRID-Fly2Human
HIRA	ASF1A	0.056571	Predicted	I2D-BioGRID-Fly2Human
SEC22B	STX5	0.302644	Predicted	I2D-IntAct-Yeast2Human
GOSR1	STX5	0.255296	Predicted	I2D-IntAct-Yeast2Human
YKT6	STX5	0.148298	Predicted	I2D-IntAct-Yeast2Human
YKT6	GOSR1	0.178633	Predicted	I2D-IntAct-Yeast2Human
NAPA	SEC22B	0.171387	Predicted	I2D-IntAct-Yeast2Human
NAPA	GOSR1	0.144574	Predicted	I2D-IntAct-Yeast2Human
NAPA	YKT6	0.083981	Predicted	I2D-IntAct-Yeast2Human
STX5	BET1	0.126583	Predicted	I2D-IntAct-Rat2Human
SEC22B	STX5	0.172225	Predicted	I2D-IntAct-Rat2Human
GOSR1	BET1	0.20089	Predicted	I2D-IntAct-Rat2Human
GOSR1	STX5	0.11082	Predicted	I2D-IntAct-Rat2Human
GOSR2	BET1	0.2695	Predicted	I2D-IntAct-Rat2Human
GOSR2	STX5	0.148668	Predicted	I2D-IntAct-Rat2Human
GOSR2	SEC22B	0.366676	Predicted	I2D-IntAct-Rat2Human
YKT6	BET1	0.214683	Predicted	I2D-IntAct-Rat2Human
YKT6	STX5	0.118429	Predicted	I2D-IntAct-Rat2Human
YKT6	GOSR1	0.18795	Predicted	I2D-IntAct-Rat2Human
VTI1A	STX5	0.254315	Predicted	I2D-IntAct-Rat2Human
SEC22B	STX5	0.151311	Predicted	I2D-BIND-Yeast2Human
GOSR1	STX5	0.204741	Predicted	I2D-BIND-Yeast2Human
YKT6	STX5	0.305408	Predicted	I2D-BIND-Yeast2Human
YKT6	GOSR1	0.462668	Predicted	I2D-BIND-Yeast2Human
STX5	BET1	0.029255	Shared protein domains	INTERPRO
STK24	STK25	0.009587	Shared protein domains	INTERPRO
GOSR2	STX5	0.040557	Shared protein domains	INTERPRO
HMGA1	HMGA2	0.41132	Shared protein domains	INTERPRO
YKT6	SEC22B	0.073803	Shared protein domains	INTERPRO
STK26	STK25	0.015847	Shared protein domains	INTERPRO
STK26	STK24	0.009587	Shared protein domains	INTERPRO
STX7	BET1	0.025598	Shared protein domains	INTERPRO
STX7	STX5	0.045061	Shared protein domains	INTERPRO
STX7	GOSR2	0.035488	Shared protein domains	INTERPRO
VTI1A	BET1	0.034144	Shared protein domains	INTERPRO
VTI1A	STX5	0.038596	Shared protein domains	INTERPRO
VTI1A	GOSR2	0.128587	Shared protein domains	INTERPRO
VTI1A	STX7	0.033774	Shared protein domains	INTERPRO
STK24	STK25	0.002837	Shared protein domains	PFAM
YKT6	SEC22B	0.121186	Shared protein domains	PFAM
STK26	STK25	0.003288	Shared protein domains	PFAM
STK26	STK24	0.002837	Shared protein domains	PFAM
STX7	STX5	0.066041	Shared protein domains	PFAM

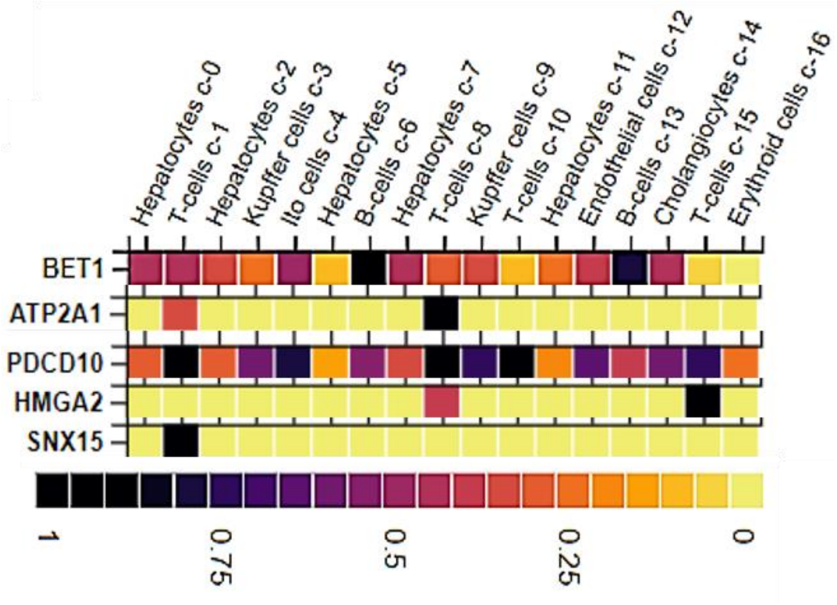

Figure S1: Heatmap of the expression levels of the CHOL-Hub genes based on single-cell RNA-sequencing dataset.
